# Supplementary material for: Impact of Plant Protein Intakes on Nutrient Adequacy in the US
Source: Nutrients. 2024 Apr 13;16(8):1158. doi: 10.3390/nu16081158 (PMC11054554; doi:10.3390/nu16081158)
Supplement: Supplementary file 1 [file nutrients-16-01158-s001.zip › nutrients-2895604-supplementary.pdf]

Supplementary Table S1. Participant flow chart

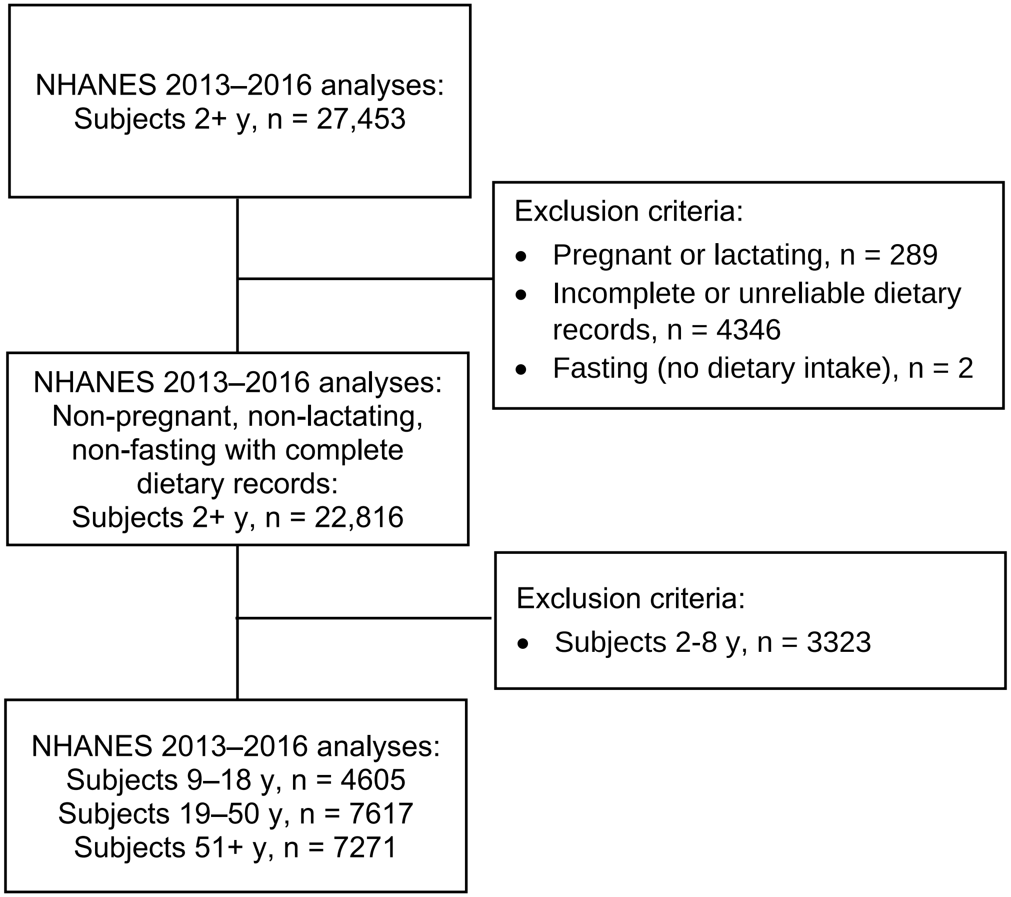

Supplementary Table S2. Percentage of adolescent age 9-18 years with nutrient intakes below Estimated Average Requirement (EAR) or above Adequate Intake (AI) across defined levels (DFL) of day 1 intake of plant protein, NHANES 2013-2018 data.

	Defined levels of day 1 intake of plant protein				Group trend	
	DFL 1 (< 25%)	DFL 2 (25% to < 50%)	DFL 3 (50% to < 75%	DFL 4 (≥ 75%)	Beta	P
Sample N	1,251	2,623	598	133		
Population N	11,566,057	23,513,743	5,680,893	1,353,083		
Nutrients with EAR[1]	% Population with intakes below EAR					
Protein	0.10 ± 0.07	0.81 ± 0.33*	11.2 ± 3.8*	47.3 ± 11.7*	8.71 ± 5.47	0.2094
Calcium	62.1 ± 2.7	61.2 ± 2.1	83.8 ± 3.5*	99.2 ± 1.4*	10.3 ± 5.5	0.1558
Copper	16.3 ± 2.0	6.74 ± 1.08*	6.73 ± 2.77*	20.4 ± 5.4	-3.04 ± 3.87	0.4894
Folate, DFE	22.4 ± 3.1	4.50 ± 0.99*	9.37 ± 3.57*	8.46 ± 4.96*	-6.90 ± 5.36	0.2882
Iron	8.63 ± 1.22	3.55 ± 0.51*	7.28 ± 2.49	10.4 ± 2.3	-0.64 ± 2.19	0.7872
Magnesium	66.6 ± 2.1	55.6 ± 1.8*	52.3 ± 4.1*	76.0 ± 8.8	-3.89 ± 5.27	0.5140
Niacin	0.24 ± 0.22	0.32 ± 0.17	6.55 ± 2.94*	14.7 ± 4.7*	3.45 ± 1.72	0.1384
Riboflavin	1.51 ± 0.54	1.12 ± 0.38	9.42 ± 3.07*	26.2 ± 6.5*	5.06 ± 3.04	0.1950
Selenium	0.04 ± 0.04	0.14 ± 0.12	2.87 ± 2.37	10.8 ± 6.0	2.06 ± 1.25	0.1971
Thiamin	7.61 ± 1.66	1.65 ± 0.45*	4.82 ± 2.55	6.86 ± 3.34	-1.42 ± 2.23	0.5696
Vitamin A, RE	38.9 ± 2.6	32.5 ± 2.3	54.0 ± 3.8*	82.8 ± 5.9*	8.77 ± 7.67	0.3357
Vitamin B_{12}	0.66 ± 0.30	1.31 ± 0.47	21.0 ± 4.6*	65.3 ± 12.2*	13.3 ± 7.4	0.1700
Vitamin B_6	1.27 ± 0.70	2.95 ± 0.84	17.0 ± 3.5*	36.1 ± 10.4*	8.60 ± 3.58	0.0956
Vitamin C	45.8 ± 2.8	34.2 ± 2.1*	32.1 ± 4.8*	57.4 ± 14.2	-3.50 ± 5.66	0.5796
Vitamin D	87.2 ± 1.8	97.4 ± 0.6*	99.9 ± 0.1*	99.6 ± 0.6*	5.92 ± 2.18	0.0726
Vitamin E, ATE	94.8 ± 1.8	81.0 ± 1.9*	68.2 ± 4.2*	79.2 ± 8.0	-10.5 ± 3.6	0.0631
Zinc	10.7 ± 2.2	16.2 ± 2.2	39.4 ± 4.9*	80.5 ± 8.1*	16.7 ± 6.3	0.0764
Nutrients with AI[2]	% Population with intakes above AI					
Dietary fiber	0.01 ± 0.01	0.33 ± 0.13*	5.80 ± 1.98*	6.65 ± 3.52	2.41 ± 1.10	0.1168
Potassium	31.3 ± 3.6	23.8 ± 2.0	17.7 ± 3.5*	1.57 ± 2.11*	-7.97 ± 1.31	0.0089
Sodium	99.8 ± 0.2	99.8 ± 0.1	97.8 ± 1.8	100 ± 6	-0.52 ± 0.58	0.4341
Vitamin K	38.6 ± 3.4	54.5 ± 2.6*	49.9 ± 5.2	29.4 ± 12.1	3.52 ± 6.72	0.6363
Choline	15.1 ± 2.2	2.81 ± 0.56*	0.30 ± 0.40*	0.00 ± 0.02*	-7.01 ± 2.66	0.0777

Data are presented as Mean ± SE. *significant differences from quartile 1 at P<0.05. [1]EAR is the average daily intake of a nutrient to meet the requirements of 50% of healthy individuals. [2]AI is the intake level assumed to ensure nutritional adequacy when insufficient data was available to establish a Recommended Daily Allowance. Abbreviations: AI, adequate intake; ATE, alpha-tocopherol equivalents; DFE, dietary folate equivalents; EAR, estimated average requirement; RE, retinol equivalents.

Supplementary Table S3. Percentage of adults age 19-50 years with nutrient intakes below Estimated Average Requirement (EAR) or above Adequate Intake (AI) across defined levels (DFL) of day 1 intake of plant protein, NHANES 2013-2018 data.

	Defined levels of day 1 intake of plant protein				Group Trend	
	DFL 1 (< 25%)	DFL 2 (25% to < 50%)	DFL 3 (50% to < 75%	DFL 4 (≥ 75%)	Beta	P
Sample N	2,404	4,081	877	255		
Population N	38,930,873	69,362,234	14,982,336	3,765,714		
Nutrients with EAR[1]	% Population with intakes below EAR					
Protein	0.17 ± 0.10	1.34 ± 0.33*	11.7 ± 2.6*	33.1 ± 15.6*	7.20 ± 3.54	0.1351
Calcium	33.2 ± 1.8	26.5 ± 1.4*	44.9 ± 2.4*	59.2 ± 12.3*	5.33 ± 5.78	0.4244
Copper	13.4 ± 1.3	6.33 ± 0.69*	5.87 ± 1.37*	7.76 ± 4.02	-3.47 ± 1.87	0.1603
Folate, DFE	28.6 ± 2.3	7.96 ± 1.27*	8.19 ± 2.30*	4.47 ± 6.45*	-10.7 ± 4.8	0.1101
Iron	12.7 ± 0.8	7.91 ± 0.44*	9.99 ± 1.62	11.5 ± 3.1	-1.45 ± 1.64	0.4424
Magnesium	61.5 ± 2.1	49.5 ± 1.3*	38.9 ± 2.3*	35.5 ± 11.1*	-10.3 ± 1.3	0.0044
Niacin	0.92 ± 0.37	0.86 ± 0.23	3.19 ± 2.30	9.69 ± 6.90	1.68 ± 1.10	0.2226
Riboflavin	3.65 ± 0.71	3.43 ± 0.58	6.19 ± 1.76	15.6 ± 5.8*	2.14 ± 1.57	0.2662
Selenium	0.08 ± 0.06	0.33 ± 0.12	3.69 ± 1.74*	20.6 ± 6.3*	3.58 ± 2.60	0.2630
Thiamin	13.0 ± 1.7	3.96 ± 0.65*	6.67 ± 2.28*	5.31 ± 5.77	-3.70 ± 2.55	0.2428
Vitamin A, RE	45.8 ± 2.2	47.3 ± 1.7	53.9 ± 3.3*	48.1 ± 11.4	2.44 ± 1.51	0.2042
Vitamin B_{12}	0.93 ± 0.47	3.50 ± 0.88*	23.9 ± 4.3*	59.3 ± 8.1*	13.4 ± 6.0	0.1128
Vitamin B_6	4.48 ± 1.25	6.85 ± 0.98	12.0 ± 4.6	14.5 ± 6.0	3.41 ± 0.54	0.0079
Vitamin C	60.3 ± 2.1	47.5 ± 1.6*	35.6 ± 4.8*	31.0 ± 6.8*	-11.4 ± 1.3	0.0029
Vitamin D	87.5 ± 2.1	96.8 ± 0.6*	98.5 ± 0.6*	99.3 ± 0.7*	5.38 ± 1.95	0.0701
Vitamin E, ATE	86.1 ± 2.0	74.6 ± 1.7*	63.7 ± 3.4*	61.7 ± 7.3*	-10.1 ± 1.5	0.0063
Zinc	7.76 ± 1.33	15.6 ± 1.2*	37.8 ± 3.1*	62.9 ± 13.4*	15.4 ± 3.8	0.0275
Nutrients with AI[2]	% Population with intakes above AI					
Dietary fiber	0.17 ± 0.09	4.19 ± 0.60*	17.3 ± 2.4*	35.4 ± 5.8*	9.21 ± 2.76	0.0446
Potassium	12.6 ± 4.2	14.5 ± 6.0	0.00 ± 6.45	8.54 ± 17.12	-3.30 ± 3.33	0.3949
Sodium	99.6 ± 0.2	99.6 ± 0.1	99.7 ± 3.7	100 ± 3	0.07 ± 0.05	0.2327
Vitamin K	43.9 ± 2.8	50.4 ± 2.4	54.7 ± 4.9	65.1 ± 10.0*	6.20 ± 0.74	0.0036
Choline	26.4 ± 2.1	7.58 ± 1.00*	1.49 ± 0.58*	0.01 ± 0.06*	-11.8 ± 3.6	0.0456

Data are presented as Mean ± SE. *significant differences from quartile 1 at P<0.05. [1]EAR is the average daily intake of a nutrient to meet the requirements of 50% of healthy individuals. [2]AI is the intake level assumed to ensure nutritional adequacy when insufficient data was available to establish a Recommended Daily Allowance. Abbreviations: AI, adequate intake; ATE, alpha-tocopherol equivalents; DFE, dietary folate equivalents; EAR, estimated average requirement; RE, retinol equivalents.

Supplementary Table S4. Percentage of adults age 51+ years with nutrient intakes below Estimated Average Requirement (EAR) or above Adequate Intake (AI) across defined levels (DFL) of day 1 intake of plant protein, NHANES 2013-2018 data.

	Defined levels of day 1 intake of plant protein				Group Trend	
	DFL 1 (< 25%)	DFL 2 (25% to < 50%)	DFL 3 (50% to < 75%	DFL 4 (≥ 75%)	Beta	P
Sample N	1,902	4,016	1,092	261		
Population N	27,705,336	60,712,458	14,677,191	3,308,016		
Nutrients with EAR[1]	% Population with intakes below EAR					
Protein	0.34 ± 0.23	2.65 ± 0.49*	11.4 ± 2.1*	50.4 ± 8.4*	9.84 ± 5.82	0.1895
Calcium	54.8 ± 2.4	56.8 ± 1.9	71.7 ± 2.5*	90.3 ± 3.5*	9.53 ± 3.54	0.0743
Copper	12.4 ± 2.1	7.80 ± 0.84*	5.18 ± 1.21*	12.1 ± 4.2	-2.27 ± 1.83	0.3030
Folate, DFE	36.3 ± 2.7	13.6 ± 1.2*	5.69 ± 1.90*	16.3 ± 5.1*	-12.2 ± 5.8	0.1271
Iron	3.31 ± 0.89	1.01 ± 0.25*	0.33 ± 0.25*	4.46 ± 2.25	-0.78 ± 1.04	0.5070
Magnesium	67.2 ± 2.3	55.4 ± 1.7*	44.0 ± 2.7*	47.1 ± 5.9*	-9.69 ± 2.36	0.0260
Niacin	1.71 ± 0.84	2.20 ± 0.41	3.70 ± 1.23	17.7 ± 5.9*	2.70 ± 2.13	0.2953
Riboflavin	2.51 ± 0.73	2.58 ± 0.41	6.56 ± 1.00*	26.1 ± 5.6*	4.26 ± 3.10	0.2630
Selenium	0.17 ± 0.15	0.53 ± 0.19	2.83 ± 1.13*	30.5 ± 12.4*	4.78 ± 4.31	0.3481
Thiamin	16.6 ± 2.1	7.41 ± 1.01*	4.78 ± 1.13*	23.2 ± 5.8	-2.83 ± 4.44	0.5696
Vitamin A, RE	32.4 ± 2.9	42.1 ± 2.4*	55.1 ± 3.2*	64.7 ± 6.3*	11.0 ± 0.7	0.0005
Vitamin B_{12}	0.99 ± 0.63	3.46 ± 0.95*	28.3 ± 3.6*	79.6 ± 8.9*	18.2 ± 8.6	0.1237
Vitamin B_6	11.8 ± 2.5	21.6 ± 1.7*	28.4 ± 3.2*	49.7 ± 7.1*	10.1 ± 2.0	0.0148
Vitamin C	55.4 ± 2.6	45.9 ± 1.9*	44.2 ± 3.3*	45.5 ± 5.9	-4.84 ± 2.24	0.1195
Vitamin D	87.1 ± 2.4	97.2 ± 0.6*	99.0 ± 0.5*	99.8 ± 0.2*	5.46 ± 2.13	0.0827
Vitamin E, ATE	85.5 ± 1.9	81.6 ± 1.5	70.5 ± 2.8*	76.6 ± 7.0	-5.56 ± 2.28	0.0929
Zinc	12.5 ± 1.9	22.0 ± 1.6*	37.6 ± 2.9*	64.6 ± 6.8*	14.3 ± 2.9	0.0161
Nutrients with AI[2]	% Population with intakes above AI					
Dietary fiber	1.67 ± 0.63	12.6 ± 1.2*	34.0 ± 3.0*	35.5 ± 5.1*	14.0 ± 2.7	0.0146
Potassium	31.9 ± 2.2	31.5 ± 1.4	28.6 ± 2.2	18.5 ± 5.1*	-2.73 ± 1.55	0.1765
Sodium	98.8 ± 0.5	98.7 ± 0.4	98.1 ± 0.7	94.1 ± 10.0	-0.82 ± 0.63	0.2826
Vitamin K	49.7 ± 3.2	54.2 ± 2.4	49.3 ± 3.1	53.3 ± 10.2	0.52 ± 1.92	0.8046
Choline	21.3 ± 2.5	5.12 ± 0.88*	0.63 ± 0.31*	0.35 ± 0.47*	-9.25 ± 3.30	0.0676

Data are presented as Mean ± SE. *significant differences from quartile 1 at P<0.05. [1]EAR is the average daily intake of a nutrient to meet the requirements of 50% of healthy individuals. [2]AI is the intake level assumed to ensure nutritional adequacy when insufficient data was available to establish a Recommended Daily Allowance. Abbreviations: AI, adequate intake; ATE, alpha-tocopherol equivalents; DFE, dietary folate equivalents; EAR, estimated average requirement; RE, retinol equivalents.
